# Association of eHealth Literacy With Lifestyle Behaviors in University Students: Questionnaire-Based Cross-Sectional Study

**DOI:** 10.2196/18155

**Published:** 2020-06-24

**Authors:** Saki Tsukahara, Satoshi Yamaguchi, Futaba Igarashi, Reiko Uruma, Naomi Ikuina, Kaori Iwakura, Keisuke Koizumi, Yasunori Sato

**Affiliations:** 1 Collage of Liberal Arts and Sciences Chiba University Chiba Japan; 2 Department of Orthopaedic Surgery Graduate School of Medical and Pharmaceutical Sciences Chiba University Chiba Japan; 3 Safety and Health Organization Chiba University Chiba Japan; 4 Clinical and Translational Research Center Keio University Hospital Tokyo Japan

**Keywords:** college student, ehealth literacy, ehealth, eHealth Literacy Scale, health literacy, lifestyle, university student

## Abstract

**Background:**

Maintenance of good health and a healthy lifestyle have significant impacts on the lives of university students. However, university students are prone to engage in risky health behaviors, resulting in impaired health status. Electronic health (eHealth) literacy is an important factor in maintaining a healthy lifestyle. However, no studies have assessed the eHealth literacy levels and the associated lifestyle behaviors among university students in Japan.

**Objective:**

The purposes of this study were to clarify the eHealth literacy level, the participant characteristics associated with eHealth literacy, and the association of eHealth literacy with lifestyle behaviors of students in a Japanese university.

**Methods:**

A questionnaire-based cross-sectional study of 3183 students at a national university in Japan was conducted. eHealth literacy was quantified using the Japanese version of the eHealth Literacy Scale (eHEALS). The association between participant characteristics (gender, school year, department of study, and living status) and eHEALS score was assessed using *t* tests. Additionally, the associations of eHealth literacy with lifestyle behaviors (exercise, smoking, alcohol consumption, etc.) were evaluated using logistic regression analyses.

**Results:**

The mean eHEALS score was 23.6/40 points. The mean eHEALS score for students in medical departments was 27.0/40 points, which was 2.9 points higher than that of nonmedical students (*P*<.001). Similarly, the graduate school participants had higher scores than the undergraduate students. The proportion of participants who exercised regularly was higher in the high eHEALS score group than in the low score group, with an adjusted odds ratio of 1.39 (*P*<.001).

**Conclusions:**

The eHealth literacy level of university students in Japan was comparable to that of the general Japanese population. Graduate students, as well as those in medical departments, had higher eHealth literacy. Furthermore, students with higher eHealth literacy had better exercise routines.

## Introduction

Healthy lifestyle behaviors, such as exercising regularly, sleeping well, and eating breakfast, have a significant impact on university student life. For example, students with healthy lifestyles achieve higher academic degrees than those without [1,2]. Moreover, an unhealthy lifestyle during university life, including smoking and alcohol use, tends to persist after graduation and is highly likely to create risk of lifestyle-related diseases in later life [3,4]. However, university students are prone to engage in risky health behaviors [5]. The transition from high school to university involves a drastic change in lifestyle, as students often start living away from their parents, developing new social networks, and having more free time than before [5]. Accordingly, a decline in university students’ health status is associated with these lifestyle changes [6-8]. Therefore, the maintenance of a healthy lifestyle is a challenge for university students.

Health literacy is defined as an individual’s knowledge, motivation, and skills to access, understand, evaluate, and apply health information [9]. Studies have shown that people with high health literacy have healthy lifestyles [10]. Therefore, improving health literacy has been identified as a public health goal and a significant health care challenge globally [11]. Currently, health information is often obtained through the internet, especially by members of the younger generation, including university students [12,13]. Collecting data through the internet is different from collecting data through books and leaflets, and it requires specific skills; people must not only be health literate but must also be able to find, understand, and appraise information using digital services and technology [14]. Accordingly, electronic health (eHealth) literacy―the ability to search for, evaluate and use health information on the internet to solve health problems―is considered to be an essential factor for university students to maintain a healthy lifestyle and good health status [15]. According to the Lily model [16], eHealth literacy consists of six core skills (or literacies), which are classified into two types: analytic skills (traditional, media, and information) and context-specific skills (computer, scientific, and health). Therefore, health literacy is one of the literacies that comprise the concept of eHealth literacy [16]. The eHealth Literacy Scale (eHEALS) is an 8-item questionnaire that was developed based on the Lily model and measures a broad overview of self-perceived eHealth literacy skills [12]. The eHEALS has been translated globally and is widely used to assess eHealth literacy levels [14]. Studies have evaluated the eHealth literacy levels of university students in different countries [15]. However, the eHealth literacy level can vary among countries. Specifically, the eHealth literacy level of Japanese people may be lower than those of people in European countries [17]. The eHealth literacy level of students in Japanese universities has not been studied.

In the general population, many personal and social background characteristics, including age, gender, household income, educational level, and occupation, are associated with health literacy levels [17,18]. Among university students, male gender, higher school year, medical study department, higher academic achievement, and higher family income are associated with higher eHealth literacy levels [19-26]. However, these studies have limitations, such as relatively small numbers of participants [19,21,23,26] and recruitment of student participants from only medical and nursing departments [21,24,25]. Therefore, a study with a larger number of participants, including both nonmedical and medical students, is required to clarify the relationship between student characteristics and eHealth literacy.

Studies have shown that people with higher eHealth literacy have healthier lifestyles than those with lower eHealth literacy in the general population [18]. Regarding university students, only a few studies have reported on the association between eHealth literacy and lifestyle behaviors, such as regular exercise, healthy eating, and regular sleep, in Taiwan, Greece, and the United States [20,22,27]. However, the numbers of participants in these studies [20,22,27] were relatively small. Furthermore, the influence of eHealth literacy on lifestyle behavior may vary depending on the country, cultural background, and degree of internet use [17,19,21].

The purposes of this study were to clarify the eHealth literacy level, the participant characteristics associated with eHealth literacy, and the association of eHealth literacy with lifestyle behaviors of students in a Japanese university.

## Methods

### Recruitment

This study was a questionnaire-based cross-sectional study performed at Chiba University, Japan. Chiba University is a national university with 13,983 students at the time of the study. Of those, 5306/13,983 (37.9%) were female and 8677/13,983 (62.1%) were male. Furthermore, 10,547/13,983 (75.4%) were undergraduate students, and 2430/13,983 (17.4%) students were studying medical sciences, including medicine, nursing, and pharmacy. Inclusion criteria were students who underwent on-campus medical examinations from April to May 2019. Exclusion criteria were students who declined to participate and who did not understand the Japanese questionnaire. Furthermore, students with incomplete answers to the questionnaire were excluded. We recruited participants during 12/19 checkup days. Of 13,983 university students, 5310 (38.0%) underwent the checkup during the 12 days. Of those, 1918/5310 (36.1%) declined to participate, and the remaining 3392 students (63.9%) answered the questionnaire. No student was excluded because of inability to understand the Japanese questionnaire. After excluding 209 students with incomplete answers, the data from 3183 students were used for analysis. The Chiba University Ethics Committee approved this study (approval number 01-02). The data were collected anonymously. No gifts or payments were given to participants for participating in this study.

### eHealth Literacy Level

The questionnaire consisted of questions on eHealth literacy, participant characteristics, and lifestyle behaviors.

The Japanese version of the eHealth Literacy Scale (eHEALS) was used to quantify eHealth literacy [12,28]. Both the original and Japanese versions of the scale have sufficient reliability and validity to evaluate the eHealth literacy of adults [12,28]. The eHEALS consisted of eight questions, including four items related to internet capability and four items related to the utilization of health information [12]. Answer options were provided with a 5-point scale from “I totally do not think so (1)” to “I think so quite (5)” for each item. The total score of eight items was calculated, with a higher score indicating a better eHealth literacy level. The participants were divided into two groups using the mean eHEALS score for statistical analysis: a high score group (≥24, n=1659) and a low score group (<23, n=1524) .

### Participant Characteristics

The participant characteristics included gender, school year, department of study, and living status. Answers for school year (first to sixth year of undergraduate studies and first to fourth year of graduate school) were dichotomized into undergraduate and graduate [20]. Answers for department of study (undergraduate: education, engineering, science, horticulture, humanities, public affairs, law, politics and economics, liberal arts and sciences, literature, medicine, nursing, pharmacy; graduate: education, horticulture, medical and pharmaceutical sciences, nursing, science and engineering, and law school) were divided into medical (undergraduate: medicine, nursing, pharmacy; graduate: medical and pharmaceutical science and nursing) and nonmedical [19,21]. Answers for living status (living alone, living with parents or partner, living in a dormitory, and other) were classified into living alone and living with others.

### Lifestyle

Lifestyle behaviors were assessed using the questions on exercise, breakfast, smoking, alcohol consumption, and hours of sleep. Answers for exercise frequency (≥3 days/week, 1-2 days/week, 1-2 days/month, none) were dichotomized into ≥1 day/week and <1 day/week for statistical analysis [18]. Answers for breakfast (every day, 5-6 days/week, 1-4 days/week, none) were divided into ≥5 days/week and <5 days/week [29]. For smoking, the answers for the three choices (yes, no, previously) were divided into nonsmoker (no, previously) and current smoker (yes) [30]. Answers concerning alcohol consumption (do not drink, 1-2 days/month, 1-2 days/week, ≥3 days/week) were categorized into <3 days/week and ≥3 days/week [31]. Answers for hours of sleep (≤6 hours, 7-8 hours, ≥9 hours) were dichotomized into sufficient (7-8 hours, ≥9 hours) and insufficient (<7 hours) [29]. Additionally, the participants’ BMI (kilograms per square meter) was recorded as a proxy of lifestyle. The answers were provided with either underweight (<18.5), normal (≥18.5, <25), overweight (≥25, <30), or obese (≥30) [32]. Overweight and obese were combined into one “overweight” category [32]. Although the answer for BMI was self-reported, we assume it was accurate because the participants’ BMIs were measured during the health examination immediately before they participated in the questionnaire survey.

### Statistical Analysis

Demographic data on the participants’ characteristics and lifestyle were expressed using descriptive statistics. Numbers and frequencies were used for categorical variables. Means and standard deviations were used for continuous variables because most of the data had a normal distribution. Participants were dichotomized into two groups depending on the characteristic (eg, undergraduate/graduate). The eHEALS scores were compared between groups using Student *t* tests. Furthermore, the associations of eHEALS scores with lifestyle behaviors were assessed using logistic regression analysis. The explanatory variable was the eHEALS score group (low/high), and the objective variables were lifestyle behaviors. The odds ratio (OR) was calculated for a healthy lifestyle in the high eHEALS group; the low score group served as the reference. Unadjusted ORs and ORs adjusted for participant characteristics (gender, school year, department of study, and living status) were expressed. Bonferroni corrections were conducted to adjust for multiplicity. Accordingly, statistical significance was set at *P*<.013 for the association between participant characteristics and eHEALS score and at *P*<.007 for the association between eHEALS score and lifestyle behavior.

## Results

### Participant Characteristics

Of the 3183 participants, 878 (27.6%) were female, and 2549 (80.1%) were undergraduate students (Table 1). Students in medical departments comprised 346/3183 (10.9%) of the survey participants.

For lifestyle behaviors, 1757/3183 (55.2%) participants exercised ≥1 day/week (Table 2). Only 140/3183 (4.3%) were current smokers, and 166 (5.2%) drank alcohol ≥ 3 days/week. Although 281/3183 (8.8%) participants were overweight, 480 (15.1%) were classified as underweight.

**Table 1:** Participant characteristics (N=3183).

Characteristic			Participants, n (%)
**Gender**			
	Male		2305 (72.4)
	Female		878 (27.6)
**School year**			
	**Undergraduate (n=2549, 80.1%)**		
		1	606 (19.0)
		2	622 (19.5)
		3	608 (19.1)
		4	613 (19.3)
		5	45 (1.4)
		6	55 (1.7)
	**Graduate (n=634, 19.9%)**		
		1	320 (10.1)
		2	271 (8.5)
		3	28 (0.9)
		4	15 (0.5)
**Department of study**			
	**Medical (n=346, 10.9%)**		
		Medicine	606 (19.0)
		Nursing	55 (1.7)
		Pharmacy	125 (3.9)
		Graduate school of medical and pharmaceutical science	14 (0.4)
		Graduate school of nursing	13 (0.4)
	Nonmedical^a^		2837 (89.1)
**Living status**			
	Living alone		1600 (50.2)
	**Living with others (n=1583, 49.7%)**		
		Living with parents	1530 (48.1)
		Living in dormitory	46 (1.4)
		Other	7 (0.2)

^a^The 2837 nonmedical students were studying education (n=436, 15.4%), engineering (n=887), science (n=257, 31.3%), horticulture (n=68, 2.4%), law, politics, and economics (n=402, 14.2%), liberal arts and science (n=101, 3.6%), literature (n=179, 6.3%), graduate education (n=28, 1.0%), graduate horticulture (n=7, 0.2%), graduate humanities and studies on public affairs (n=34, 1.2%), graduate science and engineering (n=427, 15.1%), and law (n=11, 0.4%).

**Table 2:** Lifestyle behaviors of the participants (N=3183).

Lifestyle behavior			Participants, n (%)
**Exercise**			
	**≥1 day/week (n=1757, 55.2%)**		
		≥3 days/week	657 (20.6)
		1-2 days/week	1100 (34.6)
	**<1 day/week (n=1426, 44.8%)**		
		1-2 days/month	632 (19.9)
		None	794 (24.9)
**Breakfast**			
	**≥5 days/week (n=2132, 67.0%)**		
		Every day	1651 (51.9)
		5-6 days/week	481 (15.1)
	**< 5 days/week (n=1051, 33.0%)**		
		1-4 days/week	557 (17.5)
		None	494 (15.5)
**Smoking**			
	**Nonsmoker (n=3043, 95.6%)**		
		No	2967 (93.2)
		Previously	76 (2.4)
	Smoker		140 (4.4)
**Alcohol**			
	**<3 days/week (n=3017, 96.1%)**		
		None	1208 (38.0)
		1-2 days /month	1189 (37.4)
		1-2 days/week	620 (19.5)
	≥3 days/week		166 (5.2)
**Sleep**			
	**Sufficient (n=1878, 59.0%)**		
		7-8 hours	1822 (57.2)
		≥9 hours	56 (1.8)
	Insufficient (≤6 hours)		1305 (41.0)
**BMI (kilograms per square meter)**			
	Normal (≥18.5, <25)		2422 (76.1)
	Underweight (<18.5)		480 (15.1)
	**Overweight (≥25, n=281, 8.8%)**		
		≥25, <30	230 (7.2)
		≥30	51 (1.6)

### eHealth Literacy Level

The mean eHEALS score was 23.6/40 points (SD 6.8). The mean scores for each item ranged from 2.7 to 3.1. The lowest score for was obtained for Q6: “I have the skills I need to evaluate the health resources I found on the internet,” and the highest score was obtained for Q8: “I feel confident in using information from the Internet to make health decisions.”

### Association Between Participant Characteristics and eHEALS Score

The mean eHEALS score for medical students was 2.9 points higher than that for nonmedical students (*P*<.001, Table 3). The eHEALS score was higher for graduate students than for undergraduate students (*P*=.003). However, the difference between the groups was 0.9 points. There was no difference between female and male gender (*P*=.18) or between students living alone and living with others (*P*=.02).

**Table 3:** Association between participant characteristics and eHEALS score (N=3183).

Characteristic (n)		eHEALS^a^ score, mean (SD)	*P* value
**Gender**			
	Male (2305)	23.6 (7.0)	.18
	Female (878)	23.3 (6.3)	
**School year**			
	Undergraduate (2549)	23.4 (6.8)	.003
	Graduate (634)	24.3 (6.6)	
**Department of study**			
	Medical (346)	27.0 (6.6)	<.001
	Nonmedical (2837)	23.1 (6.7)	
**Living status**			
	Living alone (1600)	23.8 (6.9)	.02
	Living with others (1583)	23.3 (6.7)	

^a^eHEALS: eHealth Literacy Scale.

### Association of eHEALS Score With Lifestyle

Overall, participants in the high eHEALS score group had a healthier lifestyle than those in the low score group (Table 4). In the high score group, 984/1659 students (59.3%) exercised regularly (≥1 day/week), while in the low score group, 773/1524 (50.7%) exercised regularly. The adjusted OR for the high score group was 1.39 (*P*<.001, Table 4). Also, 1141/1659 (68.8%) and 991/1524 (65.0%) participants in the high and low score groups, respectively, had breakfast regularly (adjusted OR 1.24; *P*=.007). Interestingly, the risk of being overweight was higher in the high score group (adjusted OR 1.49; *P*<.001). However, the number of overweight students was low in both groups, namely 176/1659 (10.6%) in the high score group and 105/1524 (6.9%) in the low score group.

**Table 4:** Association of eHEALS score with lifestyle (N=3183). OR values are for the high eHEALS score group (n=1659) relative to the low score group (n=1524).

Lifestyle behavior	Unadjusted OR^a^ (95% CI)	*P* value	Adjusted^b^ OR (95% CI)	*P* value	Model *P* value
Regular exercise	1.42 (1.23-1.63)	<.001	1.39 (1.21-1.61)	<.001	<.001
Regular breakfast	1.18 (1.02-1.37)	.02	1.24 (1.06-1.45)	.007	<.001
No smoking	1.24 (0.88-1.74)	.22	1.18 (0.8-1.67)	.36	<.001
Alcohol <3 days/week	0.80 (0.59-1.10)	.17	0.82 (0.59-1.12)	.21	<.001
Sufficient sleep	1.10 (0.96-1.27)	.17	0.91 (0.79-1.06)	.22	<.001
Overweight (n=2703)^c^	1.58 (1.23-2.05)	<.001	1.49 (1.20-2.02)	<.001	<.001
Underweight (n=2902)^d^	0.93 (0.76-1.13)	.47	.94 (0.77-1.15)	.54	.14

^a^OR: odds ratio.

^b^Adjusted for gender, school year, department of study, and living status.

^c^n=1421 (52.6%) and n=1282 (47.4%) in the high and low eHEALS score groups, respectively.

^d^n=1476 (50.9%) and n=1426 (49.1%) in the high and low eHEALS score groups, respectively.

## Discussion

### Principal Findings

We showed that the average eHEALS score of students at a Japanese national university was approximately 24 points out of 40. Several personal background characteristics, including school year and department of study, were associated with a high eHEALS score. Additionally, students with higher eHEALS scores demonstrated better exercise behaviors. To the best of our knowledge, this is the first such study of Japanese students and one of the largest studies to clarify the eHealth literacy levels and related lifestyle behaviors of university students. Our results provide important information to help university students improve their eHealth literacy and achieve healthier lifestyles.

### eHealth Literacy Level

In this study, the mean eHEALS score of the participants was 23.6/40 points. This value is comparable to that of general Japanese adults, whose mean score was 23.5 points [28,29]. However, studies from other countries have reported higher scores. For example, the mean eHEALS score of 192 students at an Iranian medical and health science university was 28.2 points [21]. In a study of 422 American college students, most of whom were undergraduate students, the mean score was 31.9 [27]. The eHEALS scores of the general population in several Asian, American, and European countries were also relatively high, ranging from 28.1-30.5 [33-36]. In addition to the lower eHealth literacy level of Japanese people, the general health literacy level, measured by using the European Health Literacy Survey Questionnaire (HLS-EU-Q47) [37], was lower in Japan than in European countries [17]. Therefore, the health literacy level in Japan appears to be low not only in university students but in Japanese people in general. There are some potential explanations for the lower health literacy of Japanese people. First, accessible and understandable public health information sites, such as the National Institutes of Health website in America [38] and the National Health Service website in Britain [39], are not available in Japan [17]. Therefore, Japanese people may have difficulty acquiring health literacy. Poor accessibility to health information can affect the eHealth literacy levels of college students in other countries as well [21]. Second, surveys by the Japanese Cabinet Office and the Ministry of Education, Culture, Sports, Science and Technology suggest that students and younger people have lower self-esteem in Japan than in other countries [40]. Because we assessed self-reported health literacy levels, the students who participated in this study may have rated their health information skills lower than the actual levels [41]. Further research is needed to clarify the cause of low health literacy in Japanese university students. Additionally, our results indicate that there is significant room for improvement in the eHealth literacy of Japanese university students [15].

### Association Between Participant Characteristics and eHEALS Score

In this study, undergraduate and graduate students in the medical sciences (ie, medicine, nursing, and pharmacy) had higher eHEALS scores than those in nonmedical departments. This result was consistent with a study of 566 Taiwanese college students, in which medical students had higher eHealth literacy in all dimensions than nonmedical students [20]. A higher eHealth literacy level in medical students was also observed in a survey of 192 Iranian university students [21]. These results are understandable because medical students are more exposed to medical and health information in their curriculum.

The graduate student participants had higher eHEALS scores than undergraduate students, although the difference in the mean score was only 0.9 points. In studies of medical and nursing students, a higher school year was associated with higher eHealth literacy [19,21,24]. Additionally, a survey of 630 Danish university students showed higher health literacy among students in master’s degree programs than among those in bachelor’s degree programs [26]. Our study showed a similar association between school year and eHealth literacy in a broader range of student populations, including medical and nonmedical students. A possible explanation for our result is that eHealth literacy levels improve during university life. Another explanation is that students with higher eHealth literacy are selected for admission to graduate programs. Further longitudinal studies are necessary to clarify this issue. Combining the results of this study and those of previous studies, education programs to improve eHealth literacy should focus on early-degree and nonmedical students.

### Association of eHEALS Score With Lifestyle

This study showed that participants in the high eHEALS score group exercised more frequently than those in the low score group. This association was significant after adjusting for participant characteristics. A relationship between higher eHealth literacy and better exercise behaviors is consistently found in Taiwanese, American, and Greek university students [20,22,27] as well as in adult internet users in Japan [29]. In this survey, 1426/3138 participants (44.8%) exercised <1 day/week. Promoting regular exercise to this population would be a fundamental part of healthy lifestyle promotion. The results of this study suggest that eHealth literacy education improves exercise behaviors.

In this study, a higher eHEALS score was associated with regularly eating breakfast. Our result was in line with previous studies, which showed that higher eHealth literacy was correlated with healthy diet behavior among American and Taiwanese college students [20,27]. People who did not eat breakfast reported suboptimal dietary behavior, such as unhealthy food choices and eating snacks [42]. Furthermore, skipping breakfast is associated with several lifestyle-related diseases [43,44] and lower academic achievement in college students [2]. Therefore, regularly eating breakfast serves as an indicator of an overall healthy lifestyle [42]. However, breakfast skipping is prevalent among young adults [45] and university students [46]. In this study, 1051/3183 (33.0%) of participants ate breakfast less than five times a week. Therefore, enhancing eHealth literacy could promote regular breakfast eating among university students.

Smoking and excessive alcohol were not associated with eHEALS score. Our results were consistent with general surveys of Japanese adults, in which the eHEALS score [29] and the general health literacy level [47,48] were not associated with smoking or alcohol consumption but were associated with exercise and balanced nutrition. Similarly, no association of eHealth literacy with smoking or alcohol was found in Greek university students [22]. This may be because the rates of habitual drinkers (166/3183, 5.2%) and current smokers (140/3183, 4.4%) were low in this study; thus, it is difficult to assess the association of eHealth literacy with alcohol and smoking [48]. Indeed, percentages of habitual drinkers and smokers have declined among young Japanese people in the past decade [49]. Another possible reason is that other environmental factors, such as friends, independent living, and family history, may affect the drinking and smoking behaviors of university students more than their eHealth literacy [50].

### Limitations

This study has several limitations. First, this study was conducted at a single national university. Therefore, the results may not apply to university students from other backgrounds. For example, the type of university (ie, public or private) could affect the students’ eHealth literacy levels [19]. Although approximately 3400 students from a wide range of school years and departments were enrolled in this study, further studies that include multiple universities are needed. Second, the participants of this study may not represent the general Chiba University student population. For example, only 878/3183 (27.6%) of participants were female, while 38% of Chiba University students are female. Third, we recruited participants who had undergone health checkups. People who undergo health checkups are more health-conscious and have healthier lifestyles than those who do not [51]; the eHealth literacy level may have been even lower if students who did not undergo health examinations had participated in the survey. Fourth, several participant characteristics that can be associated with eHealth literacy, such as academic achievement [22], family income [22], educational level of parents [26], student health history [26], and internet skills [19], were not surveyed because of the practicality limits of administering the questionnaire at the health examination. Fifth, because this study was designed as a cross-sectional study, the causal relationship of eHealth literacy with lifestyle was not clarified. Future studies that evaluate the effects of eHealth literacy education are needed to confirm the causality. Finally, we used eHEALS, which was developed in 2006, to quantify the eHealth literacy levels of the participants. However, the utilization of the internet has changed significantly since the scale was developed. Specifically, social media and mobile devices are among the most popular ways to use the internet among younger people [52]. Therefore, although this scale is a valid measurement and has been used widely, it may not fully represent the eHealth literacy levels of university students [53].

### Conclusions

The eHealth literacy level of Chiba University students was comparable to that of the general Japanese population. Graduate students, as well as those in medical departments, had higher eHealth literacy levels. Furthermore, the students with higher eHealth literacy levels demonstrated better exercise behaviors. Interventions to address eHealth literacy could help improve students’ lifestyles, although further research is warranted.

## Abbreviations

eHEALS: eHealth Literacy Scale
eHealth: electronic health
HLS-EU-Q47: European Health Literacy Survey Questionnaire
OR: odds ratio
